# Lipase-Assisted Synthesis of Alkyl Stearates: Optimization by Taguchi Design of Experiments and Application as Defoamers

**DOI:** 10.3390/molecules29010195

**Published:** 2023-12-29

**Authors:** Enoch Olvera-Ureña, Jorge Lopez-Tellez, M. Monserrat Vizueto, J. Guadalupe Hidalgo-Ledezma, Baltazar Martinez-Quiroz, Jose A. Rodriguez

**Affiliations:** 1 Area Academica de Quimica, Instituto de Ciencias Basicas e Ingenieria, Universidad Autonoma del Estado de Hidalgo, Carr. Pachuca-Tulancingo Km. 4.5, Mineral de la Reforma 42184, Hidalgo, Mexico; 2 Soluciones Quimicas Globales, S. de R.L.M.I., Rio Industrial 210, 47, Mineral de la Reforma 42186, Hidalgo, Mexico

**Keywords:** optimization, DoE, stearates, alkyl, lipase, defoamers

## Abstract

The present work proposes the optimization of enzymatic synthesis of alkyl stearates using stearic acid, alkyl alcohols (C_1_-OH, C_2_-OH, C_4_-OH, C_8_-OH and C_16_-OH) and *Candida rugosa* lipase by a L_9_ (3^4^) Taguchi-type design of experiments. Four variables were evaluated (reaction time, temperature, kU of lipase and alcohol:stearic acid molar ratio), ensuring that all variables were critical. In optimal conditions, five stearates were obtained with conversions > 90%. The obtained products were characterized by nuclear magnetic resonance (NMR). Additionally, the defoaming capacity of the five stearates was evaluated, obtaining better performance for the compound synthesized from C_8_-OH alcohol.

## 1. Introduction

Fatty acid esters are an important group of compounds that are present as reaction intermediates in the formation of amides, sulfonates and long-chain alcohols used in polymer, textile and solvent industries [1,2,3]. Stearates (C_17_H_35_COOR, with R = metal ion, carbohydrates, polyols, or alkyl groups) are essential in the production of cosmetics, drugs, lubricants and surfactants [4,5,6,7,8].

**Figure 1:**
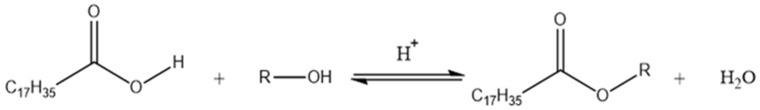
Stearic acid esterification reaction in the presence of alcohol using an acid catalyst.

The most popular method used for stearate synthesis is based on the Fisher reaction, which consists of a reaction between a carboxylic acid and an alcohol (in molar excess) in the presence of an acid catalyst such as sulfuric acid, hydrochloric acid or *p*-toluensulfonic acid (Figure 1) [9,10,11]. The reaction is performed in conventional reflux conditions (1–4 h) using benzene as a solvent and achieves yields from 58% to 75% [12,13].

The use of aromatic solvents and homogeneous acid catalysis is associated with corrosion and environmental problems [14,15]. Alternatively, the use of metallic compounds as heterogeneous catalysts for the esterification reaction has been proposed, such as SnCl_2_·2H_2_O, SiO_2_/Al_2_O_3_, zeolites and ionic exchange resins. Nevertheless, those catalysts also show some limitations, as they still require the use of organic solvents for the reaction or need to be reactivated [14,15,16].

Current trends are looking to reduce the use of solvents and catalysts that are harmful to the environment and, therefore, the use of enzymatic catalysis has been proposed as an environmentally friendly alternative. Lipases are enzymes used in ester hydrolysis reactions in aqueous media, to form carboxylic acid and alcohols [17]. However, when lipase is added to the mixture of carboxylic acid and alcohol, the esterification reaction proceeds and, hence, it is considered as an alternative to synthesize alkyl stearates. Formation of lipase-catalyzed esters has increased in recent years, and lipases from *A. niger*, *R. delemar*, *G. candidum* and *Penicillium cyclopium* were described to synthesize esters using fatty acids and various primary and secondary alcohols [18], with variable experimental conditions being reported.

The objective of the design of experiments (DoE) is to reduce the necessary number of experiments in order to obtain an optimal value and provide information on the influence of the factors involved [19]. The use of DoE is a valuable option to reduce experimentation, it has been widely used in enzymatic esterification. Factorial design was used to evaluate the effect of variables in esterification reactions using *Mucor miehei* lipase [20], Novozym-435 [21] and *Thermomyces lanuginosus* lipase [22], 2–4 variables were evaluated (temperature, lipase amount, reaction time and alcohol:acid molar ratio) in factorial designs 4–16 experiments, followed by optimization by Central composite designs for two variables (13 additional experiments) [23]. Taguchi-type design is a robust tool to optimize processes with a minimum of experimentation, this strategy was described to optimize four variables (three levels for each one) with nine experiments, in the enzymatic synthesis of ethyl levulinate [24].

Defoamers are a group of compounds or mixtures that can break foam formation in a system, while antifoamers inhibit foam production [25]. Defoamers are composed of hydrophobic particles which reduce the foam stability [26]. They are classified into three types: oil-based defoamers (mineral and silica oils), alkoxylated copolymers, and emulsified hydrophobic compounds [27]. In all cases, they produce a physical, chemical, or mechanical change in surface tension, elasticity, viscosity, or electrostatic charge [28]. The most common compounds used as defoamers are vegetable oils, paraffinic and naphthalenic oils, fatty acids, esters and alcohols [29].

Considering the hydrophobic nature of alkyl stearates, they have similar characteristics to other compounds used in the defoamers industry and, so, they can be considered good candidates for applications in process control of different industries. The present work proposes the optimization of the enzymatic synthesis conditions for the production of alkyl stearates from stearic acid, alkyl alcohols and *Candida rugosa* lipase, using a Taguchi-type design of experiments followed by the application of alkyl stearates as defoamer agents.

## 2. Results

Synthesis conditions were evaluated using a Taguchi-type design of experiments, and the evaluated conditions were: alcohol:stearic acid molar ratio (5.0:1.0, 10.0:1.0 and 15.0:1.0), temperature (40, 50 and 60 °C), time (1, 3 and 5 days) and lipase units (7.0, 21.0 and 35.0 kU). All of these control variables are critical in transesterification and esterification reactions with enzymatic catalysis [12,30]. The addition of a molar excess of alcohol promotes product formation [31]. Rodrigues et al. evaluated alcohol molar ratios between 3:1 and 12:1 in biodiesel formation using Lipozyme TL-IM, Lipozyme RM-IM and Novozym 435, with methanol, ethanol, propanol and butanol. Yield value depends on the alkyl length, and a higher yield was observed for methanol in a molar ratio of 5:1, while for butanol the best ratio was 9:1 [32]. Temperature represents an important parameter for the correct functioning of the lipase, as enzyme denaturalization can be observed for temperatures higher than 60 °C. During the synthesis of octyl oleate using Lipozyme 10,000 from *Rhizomucor miehei* as a catalyst, temperatures from 20 to 60 °C were evaluated and a higher conversion was obtained for temperatures between 45 and 60 °C [33]. The reaction time may be modified depending on lipase type, and the activity differences between lipases from *Candida rugosa* and *Candida antarctica* were studied by Tsitsimpikou et al. [34]. Both lipases were used in the esterification of lauric acid and different types of carbohydrates. The lipase from *Candida rugosa* presented longer reaction times (3 to 5 days) than that of *Candida antarctica* (1 to 3 days), thus a larger amount of *Candida rugosa* lipase was required. Similar behavior was observed for octyl oleate synthesis using *Candida antarctica* lipase B [35].

Once the levels of the control variables were selected, alkyl stearates conversion (%) was defined as the output variable. In order to evaluate four variables at three levels, a Taguchi parameter design L_9_ (3^4^) was used. The design matrix and average conversion (%) are shown in Table 1.

**Figure 2:**
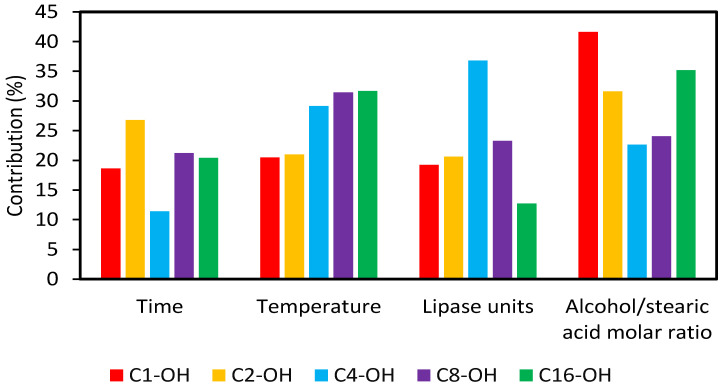
Contribution of the variables in the performed Taguchi-type L_9_ (3^4^) design of experiments.

Syntheses were performed by triplicate and results were statistically analyzed by ANOVA TM-v2.5 program, to know the contributions of each variable and the subsequent selection of optimal values (Figure 2). In all cases, variables were critical if F_exp_ > F_crit_. For C_1_-OH, C_2_-OH and C_16_-OH, the variable that exerted the greatest contribution was alcohol:stearic acid molar ratio. For C_4_-OH and C_8_-OH, critical variables were lipase units and temperature, respectively. The obtained results are consistent with those described by Deng et al. [36], where methyl-, ethyl- and butyl-stearates were synthesized by enzymatic catalysis, using *Candida* sp. 99-125 lipase, and where alcohol:stearic acid molar ratio was found to be the most important variable in the process. On the other hand, it has been described that temperature and lipase units were the most important variables in cetyl esters synthesis [37].

**Figure 3:**
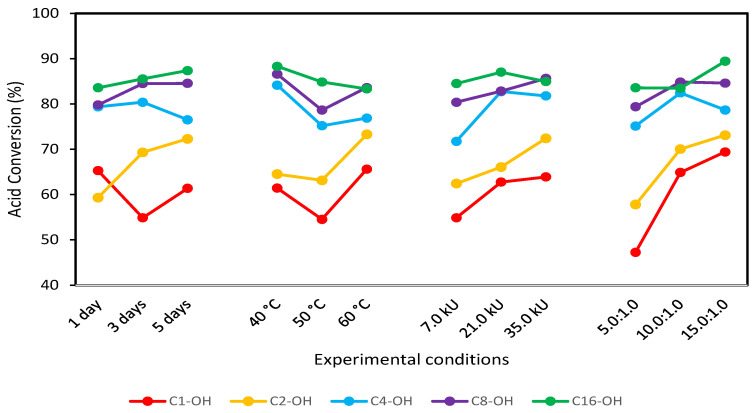
Mean graph obtained with the L_9_ (3^4^) Taguchi-type design of experiments for the esterification reaction using C_1_-OH, C_2_-OH, C_4_-OH, C_8_-OH and C_16_-OH.

From the obtained results, optimal reaction conditions were selected (Figure 3) using as the criteria “most is better”. Variables combination and the obtained conversions are shown in Table 2, confirming that conversion increases with alcohol hydrophobicity. It has been described that methanol and ethanol, in lipase-based esterification reactions, inhibit lipase enzymatic activity. *Candida antarctica* lipase B inhibition was evaluated in vinyl acetate esterification using methanol [38], and higher catalytic activity was found for lower methanol concentrations, while lipase inhibition was marked for higher methanol concentrations [39]. Similar behavior was described in ethyl butyrate synthesis using *Candida cylindraceae* as a catalyst, where the loss of lipase activity was marked for higher concentrations of ethanol [40]. Enzyme inhibition is also observed in the presence of long-chain alcohols; however, the yield is higher compared with methanol and ethanol reactions. *Candida* sp. 99-125 was evaluated in oleic acid reaction using cetyl alcohol, and the obtained yields were between 91% and 94%, despite observing enzymatic inhibition [41].

**Figure 4:**
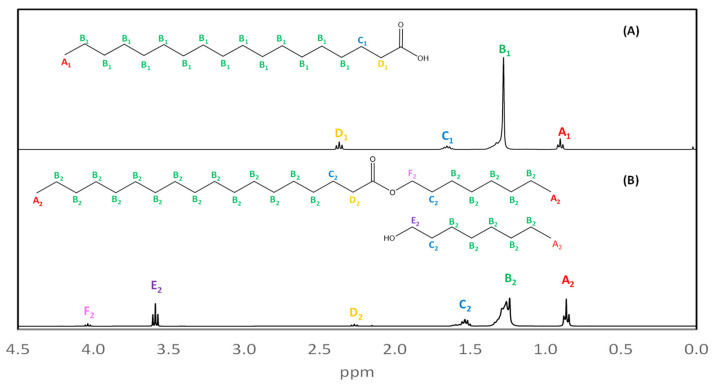
(**A**) ^1^H NMR spectrum obtained for stearic acid used as raw material. (**B**) ^1^H NMR spectrum obtained for C_8_-OH stearate, whose displacement adjacent to oxygen protons to ester group was found at 4.02 ppm.

Products can be identified using the signal from methylene protons adjacent to oxygen bonded to carbonyl (D_1_ and D_2_, in yellow), which are shifted to lower frequencies than the ester signals. Stearate from C_8_-OH ^1^H NMR spectra (Figure 4B) was compared to stearic acid used as raw material (Figure 4A). A_1_ and A_2_ signals (in red) correspond to methyl protons from both compounds, B_1_ and B_2_ signals (in green) correspond to methylene alkyl chain, C_1_ and C_2_ peaks (in blue) represent the protons adjacent to carbons near the functional group, and D_1_ and D_2_ signals are from methylene from α-carbons adjacent to the carbonyl. F_2_ (in pink) signal confirms the C_8_-OH stearate formation because this peak corresponds to methylene protons adjacent to an oxygen atom from an ester group and it can be found at higher frequencies. However, E_2_ (Figure 4B), marked in purple, corresponds to methylene adjacent to -OH present in octanol, indicating its presence in the mixture.

From the ^1^H NMR spectra, a comparison was made between the raw material (stearic acid) and the synthesized product. The presence of alkyl stearates can be confirmed in different experiments from signals of displacement caused by hydrogens adjacent to carbonyl-bonded oxygen observed at 3.60–4.13 ppm which is not found in a carboxylic acid spectrum. This characteristic displacement can be observed in C_1_-OH to C_16_-OH synthetized esters and can be distinguished from the signals attributed to alcohols which are obtained at 3.48–3.62 ppm. In all cases, characteristic signals of alkyl stearates were observed.

**Figure 5:**
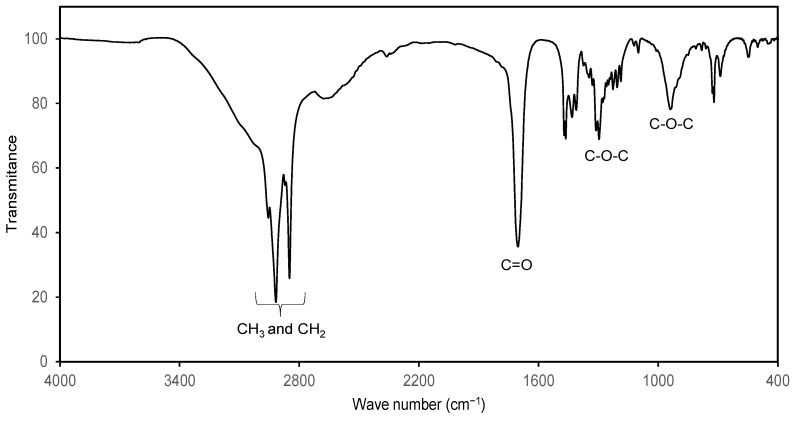
FTIR spectrum of C_8_-OH stearate and characteristic signals of the ester group.

In order to confirm the ^1^H NMR study, the products were characterized by FTIR. Figure 5 shows the spectrum of C_8_-OH stearate where the characteristic tension signals of methyl and methylene can be observed in an interval of 3000–2800 cm^−1^, at 1700 cm^−1^ tension signal of the carbonyl group is observed and, in the intervals of 1150–1400 and 850–1000 cm^−1^ there are the tension signals of the C-O-C bonds that corroborate the presence of an ester group. On the other hand, no signs of hydroxyl groups (from alcohol and carboxylic acid).

**Figure 6:**
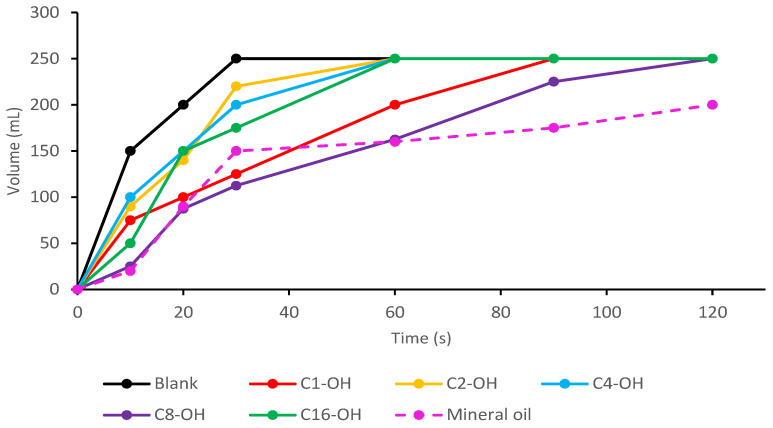
Defoaming capacity evaluation for the synthesized alkyl stearates and comparison to mineral oil.

Under the optimized conditions previously described, defoaming evaluation was made in duplicate for each alkyl stearate (Figure 6). Mean results showed that the prepared mixtures can control the foam in a short period, compared to other developed defoamers such as carbohydrate esters, fatty acid alcohols, ethoxylates or nitrogen compounds [8,24,26]. On the other hand, alkyl stearates can control foam production if they are mixed with other defoamers to achieve better performance.

From total area calculation for the Simpson rule to numeric integration, the percentage (%) of foam elimination was calculated for each one of the prepared mixtures, using as a reference the volume reached over time (Figure 6). It was determined that the defoamer prepared from C_8_-OH stearate eliminated 26% of foam in water, while C_1_-OH stearate eliminated 18%, C_16_-OH eliminated 15%, and C_2_-OH and C_4_-OH only eliminated 9% of foam in water. This behavior is consistent with the definition of defoamer, due to the alkyl stearates control foam formation but they do not inhibit it [25]. Estimated log *p* value of each stearate were: C_1_-OH = 8.32, C_2_-OH = 8.56, C_4_-OH = 9.04, C_8_-OH = 9.58 and C_16_-OH = 10.18 [42]. C_8_-OH stearate presented better results due to its intermediate hydrophobicity, which promotes the interaction with both hydrophilic and lipophilic compounds. The defoaming capacity was compared to mineral oil emulsion (Figure 5), which is commonly employed for this purpose. It was found that mineral oil eliminated 28% of foam in water which was similar to C_8_-OH stearate emulsion (26%).

## 3. Materials and Methods

### 3.1. Materials and Instruments

*Candida rugosa* lipase (Type VII ≥ 700 units/mg) was obtained from Sigma-Aldrich (St. Louis, MI, USA). Methanol (C_1_-OH), ethanol (C_2_-OH), butanol (C_4_-OH) and octanol (C_8_-OH) were obtained from J.T. Baker (Phillipsburg, NJ, USA). Cetyl alcohol (C_16_-OH) was obtained from BASF (Ludwigshafen, Germany). Stearic acid (C_17_H_35_COOH) used throughout the work was technical grade. An incubator brand Boekel Scientific model 132000 (Boekel Scientific, Feasterville-Trevose, PA, USA) was used to maintain the temperature of the reaction, a nuclear magnetic resonance (NMR) 400 MHz Bruker equipment (Bruker, Billerica, MA, USA) and a Fourier transform infrared spectroscopy (FTIR) Perkin-Elmer spectrum GX 59750 (Perkin-Elmer, Waltham, MA, USA) were used to characterize the products.

### 3.2. Synthesis of Alkyl Stearates

In order to optimize the synthesis of alkyl stearates, the variables time, temperature, alcohol:stearic acid molar ratio and enzymatic units of *Candida rugosa* lipase were evaluated. In 5-mL glass ampoules, 1.0 g (3.51 mmol) of stearic acid, *Candida rugosa* lipase (7.0–35.0 kU) and the corresponding alkyl alcohol (R-OH:C_17_H_35_COOH) in a molar ratio of 5.0:1.0 to 15.0:1.0 [31] were added. Subsequently, the ampoules were sealed and kept at a constant temperature (40–60 °C) in an incubator to react for 1–5 days [12].

Once the reaction time was concluded, vials were heated in a water bath at 60 °C to maintain the products in liquid phases, with three phases being observed, namely a solid phase (lipase) and two liquid phases. The superior liquid phase was the non-reactive alcohol and the middle liquid phase was the alkyl stearate, which was aspirated (using a pipette) and stored in a vial to calculate the acidity index (*AI*) and the reaction conversion, by the following procedure: 40 mg of alkyl stearate was weighted and diluted in 10 mL of heated methanol (40 °C), and the solution was titrated with KOH (0.1 M) standardized solution, using phenolphthalein as indicator. The acidity of samples (mgKOH g^−1^) was calculated using Equation (1) and subsequently the alkyl stearate obtained conversion was calculated using Equation (2).

(1) $$AI=\frac{\left(56.1)(C_{KOH})(V_{KOH}\right)}{m}$$

(2) $$Conversion\;\left(\%\right)=\frac{\left({AI}_{i}-{AI}_{f}\right)}{{AI}_{i}}\times 100$$

where: *AI* = acidity index, *C_KOH_* = KOH molarity (mol/L), *V_KOH_* = KOH volume (mL) used for titration, *m* = analyte mass (g), *AI_i_* = Initial acidity index and *AI_f_* = Final acidity index [11].

Additionally, the obtained products were analyzed by ^1^H nuclear magnetic resonance, using CDCl_3_ as solvent and acquiring the spectra at room temperature and by Fourier transform infrared spectroscopy in KBr tablet with a range of 400–4000 cm^−1^.

### 3.3. Evaluation of Emulsion Performance

Synthetized stearates were dispersed in water (oil-in-water emulsion) for their application as defoamers. Stock emulsions of each alkyl stearate were prepared with a non-volatile content of 20.0% (*w*:*w*). The hydrophobic phase, composed of the alkyl stearate (20.0%) and Span 60 (1.0%), was prepared in a container while the hydrophilic phase was prepared in parallel, in a separated container, by mixing lauryl ether sodium sulfate (4.0%), Tween 80 (1.0%) and water (74.0%). Both phases were placed in a water bath at 60 °C for 10 min with constant stirring. The hydrophobic phase was added to the hydrophilic phase and the mixture was kept stirring in two steps, the first 5 min at 500 rpm followed by a second stirring at 10,000 rpm for 1 min (Turrax IKA T18 equipment, IKA, Staufen, Germany). Emulsions were cooled at constant stirring to achieve room temperature and left stored until use. The emulsions are stable at room temperature for six months without observing any phase separation.

Defoaming performance of each alkyl stearate emulsion was evaluated by the Ross-Miles model [43], using a foaming aqueous solution containing sodium dodecylbenzensulfonate (0.01% *w:w*) and xanthan gum (0.015% *w:w*). In a 500 mL cylinder, 250 mL of foaming solution and 5 mL of a 100-fold diluted emulsion were mixed. The mixture was homogenized, and an air flux (450 mL/min) was introduced through the lower part. The generated foam volume was measured at each 30 s, and the time vs. foam volume was plotted to determine the area under the curve. The defoaming performance was determined by comparing the areas obtained with and without alkyl stearate addition.

## 4. Conclusions

Alkyl stearates C_1_-OH, C_2_-OH, C_4_-OH, C_8_-OH and C_16_-OH were synthesized in optimal conditions from stearic acid and the corresponding alkyl alcohols, under enzymatic catalysis (*Candida rugosa* lipase) by Taguchi-type L_9_ (3^4^) design of experiments. Critical variables were identified for each obtained product and their contributions were calculated. It was observed lipase inhibition using C_1_-OH and C_2_-OH alcohols, decreased the conversion. Additionally, characterization of the products was made by ^1^H NMR and FTIR to confirm the alkyl stearates formation. Subsequently, the defoamer properties of the five obtained products were evaluated, and the best performance was observed for the C_8_-OH compound since its log *p* is at a midpoint which allows it to interact with hydrophobic and hydrophilic compounds. The results showed that these compounds have limited performance as defoamers. Nevertheless, by using enzymatic catalysis, the implemented synthesis does not require the use of harmful organic solvents such as benzene or the use of complex methodologies. Moreover, the obtained alkyl stearates can be applied in the production of cosmetics or lubricants. The use of a design of experiments as L_9_ (3^4^) Taguchi-type optimized the system in nine experiments is a useful alternative to evaluate systems for industrial processes.

## Figures and Tables

**Table 1:** Levels and factors chosen for L_9_ (3^4^) design optimization.

Experiment	Time (Days)	Temperature (°C)	Lipase (kU)	Alcohol:Stearic Acid Molar Ratio	Mean Reaction Conversion (%RSD, *n* = 3)				
					C_1_-OH	C_2_-OH	C_4_-OH	C_8_-OH	C_16_-OH
1	1	40	7.0	5:1	47.2 (2.2%)	41.0 (2.4%)	74.1 (1.4%)	77.2 (0.8%)	83.4 (1.5%)
2	1	50	21.0	10:1	65.8 (1.6%)	57.6 (1.8%)	83.5 (1.2%)	78.3 (2.7%)	81.4 (1.3%)
3	1	60	35.0	15:1	82.6 (0.5%)	77.2 (1.4%)	80.4 (0.8%)	84.8 (0.9%)	84.8 (0.8%)
4	3	40	21.0	15:1	66.9 (0.6%)	72.1(1.5%)	89.6 (1.3%)	89.6 (1.2%)	93.8 (1.1%)
5	3	50	35.0	5:1	38.9 (2.7%)	61.7 (1.7%)	76.2 (1.4%)	79.3 (1.4%)	82.4 (1.3%)
6	3	60	7.0	10:1	58.7 (0.8%)	74.1 (1.4%)	75.2 (0.5%)	84.5 (1.2%)	80.3 (1.3%)
7	5	40	35.0	10:1	70.0 (1.5%)	78.3 (1.3%)	88.6 (1.2%)	92.8 (1.1%)	87.6 (0.9%)
8	5	50	7.0	15:1	58.6 (1.0%)	70.0 (1.5%)	65.8 (1.6%)	79.3 (0.7%)	89.7 (1.0%)
9	5	60	21.0	5:1	55.4 (2.0%)	68.5 (1.6%)	75.0 (1.4%)	81.5 (1.3%)	84.8 (0.7%)

**Table 2:** Average conversion (%) of the alkyl stearates synthetized under optimal conditions.

Alkyl Stearate	Time (Days)	Temperature (°C)	Lipase (kU)	Alcohol:Stearic Acid Molar Ratio	Conversion (%RSD *n* = 3)
C_1_-OH	1	60	35.0	15:1	90.5 (0.6%)
C_2_-OH	5	60	35.0	15:1	91.9 (0.7%)
C_4_-OH	3	40	21.0	10:1	94.7 (0.3%)
C_8_-OH	3	40	35.0	10:1	96.4 (0.3%)
C_16_-OH	5	40	21.0	15:1	96.8 (0.5%)

## Data Availability

Data are contained within this article.
